# The effect of erectile dysfunction on quality of life in male kidney transplant recipients

**Published:** 2014

**Authors:** Wei-Gang Wang, Ping Li, Yi-Shu Wang, Gang Wang, Yuan-Tao Wang, Hong-Lan Zhou

**Affiliations:** 1 Dr. Wei-Gang Wang, Second Department of Urology, First Hospital of Jilin University, Changchun 130021, China.; 2 Dr. Ping Li, Department of Rheumatology, China-Japan Union Hospital of Jilin University, Changchun 130021, China.; 3 Dr. Yi-Shu Wang, Department of Pathology, School of Basic Medical Sciences, Jilin University, Changchun 130021, China.; 4 Dr. Gang Wang, Second Department of Urology, First Hospital of Jilin University, Changchun 130021, China.; 5 Dr. Yuan-Tao Wang, Second Department of Urology, First Hospital of Jilin University, Changchun 130021, China.; 6 Dr. Hong-Lan Zhou, Second Department of Urology, First Hospital of Jilin University, Changchun 130021, China.

**Keywords:** Kidney, Male, Renal transplant, Erectile dysfunction, Quality of life

## Abstract

***Objective*** ***:*** To assess how erectile dysfunction (ED) affects the quality of life in male kidney transplant recipients.

***Methods:*** We randomly selected 150 cases of married male kidney transplant recipients. Using the International Index of Erectile Function (IIEF-5) Questionnaire, we divided our research subjects into ED group (n=63) and non-ED group (n = 87). The Short-Form health survey (SF-36) was used to evaluate the quality of life of the recipients. Hamilton Anxiety Rating Scale was used to compare the mental health status of the two groups.

***Results:*** No significant differences (P > 0.05) were observed between the ED and non-ED groups in physical functioning (PF), role-physical (RP), or bodily pain (BP). However, the ED group exhibited a lower score (P < 0.05) than the non-ED group in general health (GH), vitality, social functioning (SF), role emotional (RE) and mental health (MH). There were 13 cases in the ED group with anxiety disorders (20.6%), which was clearly more than in the non-ED group (3.4%, *P *< 0.05).

***Conclusion:*** Erectile dysfunction is an important factor in the quality of life of male kidney transplant recipients.

## INTRODUCTION

Since the first case of renal transplantation in the last century, researchers have focused on prevention of kidney graft rejection and survival of transplant recipients and grafts.^1^ In recent years, with the application of new immunosuppressive agents, survival rates of both kidney transplant recipients and grafts have been significantly improved. Therefore, the search for methods to improve the quality of life of transplant recipients is gaining more attention.-

Human health is a combination of physical, psychological and social health. Sexual function plays an important role within human health. Male erectile dysfunction (ED), the inability to achieve and maintain an erection adequate to perform sexual intercourse, is an important issue worldwide and is reported to occur in 5% to 69% of men in community-based studies.^5^^,^^6^ It is more common in patients with chronic kidney disease (CKD) and those on peritoneal dialysis and hemodialysis (HD), and is found to occur in more than 80% of those patients, while an even greater percentage of them complain of decreased libido and a marked decline in the frequency of intercourse.^7^^,^^8^ Studies have shown that kidney transplantation can improve sexual function in some recipients.^9^ However, erectile dysfunction is still common in male kidney transplant recipients. Studies have shown that 48% to 56% of renal transplant recipients had ED.^10^ Plausible reasons for impaired erectile function in transplant recipients are recipient co-morbidities, the transplant operation, adverse effects of medication, relationship problems and changes in mental health due to psychological and physical stress. In clinical practice, the attention given to sexual problems in this group of patients is low. It is still unclear how ED will affect quality of life in male kidney transplant recipients. To investigate this problem, we took advantage of the more than 1,500 cases of kidney transplant recipients that passed through our center and randomly selected 150 cases of married male recipients.

The Short Form health survey (SF-36) is a commonly and internationally used form for assessment of health-related quality of life.^11^ It contains eight scales, which include physical functioning, physical role functioning, bodily pain, general health perception, vitality, social role functioning, emotional role functioning and mental health.^12^ The first four scales evaluate physical health and the latter four scales evaluate psychological health. The score for each scale is converted into a percentile so that each scale is assigned a score from 0 to 100. SF-36 has been used to assess the quality of life in renal transplant recipients and the higher the score, the higher the quality of life.^13^^,^^14^

Psychological status has been shown to be associated with quality of life in renal transplant recipients.^15^^,^^16^ The Hamilton Anxiety Rating Scale (HAM-A) is a psychological questionnaire used by clinicians to rate the severity of a patient's anxiety. It was originally published by Hamilton in 1959 and remains widely used by clinicians.^17^ It contains 14 symptom-oriented questions. Each of these symptoms is given a severity rating, from not present (scored as zero) to very severe (scored as 4). The clinician must choose the possible responses to each question by interviewing the patient and observing the patient's symptoms. A total score of 0-17 is considered mild, 18-25 mild to moderate, and 26-30 moderate to severe. Using the SF-36 and Hamilton Anxiety Scale score, we evaluated the physical and mental health status of our research subjects. By analyzing the data and associating health status with ED, recipients with ED were found to have lower life quality scores and more anxiety than those without ED. The intention is that our study will draw more attention to the problem of life quality in male kidney transplant recipients, especially with regard to their sexual function. Treatment of ED in male recipients will significantly improve their health condition and quality of life.

## METHODS

***Research subjects: ***For the present study 150 cases were selected from the kidney transplant recipients that were enrolled at our center between January 1, 2000 and January 1, 2012. Inclusion criteria were: [1] male; [2] married; [3] 20 to 45 years old; [4] more than 6 months post-surgery; [5] serum creatinine ≤ 200 mmol/L; [6] first time recipient of a kidney transplant; [7] no complications; [8] capable of communication and completing the questionnaire; and [9] consistency with the principles of voluntary participation and approval by the hospital ethics committee. Exclusion criteria were: [1] severe liver dysfunction; [2] severe psychological and mental disorders; and [3] severe heart dysfunction.

***Grouping of subjects: ***Subjects with ED questionnaire-5 (IIEF-5) scores less than 21 points were diagnosed as having ED. Based on this standard, all research subjects were divided into the ED group (63 cases) and non-ED group (87 cases) (Table-III).

***Data collection: ***An in-house questionnaire was used to collect general information on the research subjects, such as age, time after surgery, immune suppression regimen, arterial anastomosis, educational level, employment status, marital status, income, and medical expenditure. The SF-36 was used to evaluate quality of life in research subjects. Hamilton Anxiety Scale score was used to assess the severity of anxiety in research subjects. A score greater than 14 is defined as having anxiety disorders. All questionnaires were distributed by the same physician, patients were provided with standard instructions, forms were completed by recipients either orally or in written form, and were collected within 20 to 30 minutes.

***Study approval: ***The study was reviewed and approved by the Ethics Committee of Bethune First Hospital of Jilin University. Informed written consent was obtained from each subject.

***Statistical analysis: ***Analysis of data was done with SPSS 13.0 software (SPSS Inc.; Chicago, IL, USA). Results are shown as mean±SD. t-test or Chi-square test was used to compare differences between groups. *P *< 0.05 was considered as statistically significant.

## RESULTS

***General information on subjects in the ED and non-ED groups: ***Validated in-house questionnaires regarding general information were collected from the 150 subjects who met the inclusion criteria. The ED and non-ED groups were not significantly different in any aspects of their general health conditions, including age, time after surgery, immune suppression regimen, arterial anastomosis, educational level, employment status, marriage status, income, or medical expenditure (*P *> 0.05; Table-I).

***ED group had lower life quality score than non-ED group: ***Life quality of ED and non-ED groups was evaluated by using SF-36. The ED group was not significantly different from the non-ED group in physical functioning, physical role functioning and bodily pain (*P* > 0.05). However, the ED group had a clearly lower score than the non-ED group in general health, vitality, social role functioning, emotional role functioning and mental health (*P *< 0.05; Table-II).

Subjects in the ED group consciously felt that they tended to get sick, and claimed that their health status was deteriorating. They were more likely to reduce their working hours, activity and social interactions due to negative emotions, such as depression or anxiety. Some patients became very sensitive, emotional and susceptible to fatigue.

***ED group subjects were more anxious than non-ED group subjects: ***To further explore the mental health status of our research subjects, we used the Hamilton Anxiety Scale. A score greater than 14 was defined as having an anxiety disorder. There were 14 subjects in the ED group who were deemed to have an anxiety disorder (20.6%) and the average score for patients in the ED group was 10.6 ± 9.3 (Range: 1-22). In the non-ED group, the percentage of patients with anxiety disorders was 3.4% and the average score was 8.9 ± 7.2 (Range: 0-18). Therefore, the ED group had more patients with anxiety disorders and had a higher average score than the non-ED group, indicating they were generally more anxious.

## DISCUSSION

The success of kidney transplantation has more recently resulted in a focus on quality-of-life issues.^4^ The World Health Organization defines ‘Quality of Life’ as the perception of an individual of their position in life in the context of the culture and value systems in which they live and in relation to their goals, expectations, standards and concerns.^18^ It is a broad-ranging concept affected in a complex way by physical health, psychological state, level of independence, social relationships, personal beliefs and relationship to salient features of their environment. Currently, it is believed that kidney transplantation significantly improves the quality of life of recipients.^19^^,^^20^ In clinical practice, however, we have seen transplant recipients who considered that their quality of life did not improve. For example, some kidney transplant recipients refrained from work because they worried about infectious diseases due to their weakened immune system. Some recipients became afraid of social interactions because of the change of their facial appearance.

Sexual function plays a very important role in the life of male recipients. Sexual function was gradually restored in some of the recipients, but 30% to 50% of the recipients still suffer from ED.^21^^,^^22^ Besides dysfunction of hypothalamic-pituitary-gonadal axis, the well-known causal factor of ED, many other factors can influence sexual function of male renal transplant recipients.^9^^,^^23^ EI-Bahnasawy and colleagues evaluated 400 male patients after renal transplantation and found 35.8% of them had ED.^24^ Multiple factors were significantly associated with ED, such as age, hemoglobin level and presence of diabetes mellitus and/or peripheral neuropathy. Rebollo and colleagues also showed that various factors, including longer time on dialysis prior to transplantation, lower diastolic pressure and peripheral artheriopathy, negatively impact sexual function of male renal transplant recipients.^25^ In our study, 42% of the recipients had ED, and among those 49.2% had severe ED. However, whether ED has a large influence on recipient quality of life is still unclear. To answer this question, the present study was conducted to compare quality of life in ED and non-ED male kidney transplant recipients.

**Table-I:** General information for ED and non-ED groups (*x *± *s*).

*Group*	*Number of cases*	*Age * *(years)*	*Time after transplantation * *(years)*	*Immunosuppressive regimen * *(cases)*		*Renal artery vascular anastomosis * *(cases)*		*Workers (cases* *)*
				*CsA-based*	*Tac-based*	*internal iliac* *artery anastomosis*	*external iliac* *artery anastomosis*	
ED	63	45.2 ± 11.5	7.3 ± 4.5	34 / 54.0%	29 / 46.0%	43 / 68.3%	20 / 31.7%	52 / 82.5%
Non-ED	87	39.8 ± 13.6	6.8 ± 5.3	51 / 58.6%	36 / 41.4%	61 / 70.1%	26 / 29.9%	74 / 85%
*P * *=*	-	> 0.05	> 0.05	> 0.05	> 0.05	> 0.05	> 0.05	> 0.05
Groups	Educational status (cases)			Divorced (cases)	Monthly income (cases)			Medical insurance (cases)
	Below junior school	Below high school	Above college		< 1000	1000-5000	> 5000	
ED	16 / 25.4%	29 / 46.0%	18 / 28.6%	4 / 6.3%	17 / 27.0%	34 / 54.0%	12 / 19.0%	47 / 74.6%
Non-ED	20 / 23.0%	41 / 47.1%	26 / 29.9%	6 / 6.9%	21 / 24.1%	48 / 55.2%	18 / 20.7%	67 / 77.0%
*P * *=*	> 0.05	> 0.05	> 0.05	> 0.05	> 0.05	> 0.05	> 0.05	> 0.05

**Table-II:** Quality of life scores of ED and non-ED groups (*x *± *s*).

*Groups*	*Number of cases*	*Physical function*	*Physiological functions*	*Bodily pain*	*General health*	*Vitality*	*Social function*	*Emotional role function*	*Mental health*
Non-ED	87	80.2 ± 13.7	38.5 ± 33.4	75.4 ± 16.3	62.7 ± 16.4	79.3 ± 12.5	57.6 ± 20.3	77.5 ± 21.2	78.6 ± 14.3
ED	63	78.3 ± 14.1	37.8 ± 34.2	73.6 ± 16.6	47.4 ± 17.3	56.8 ± 13.7	42.3 ± 21.6	60.7 ± 23.6	62.4 ± 11.7
*t*	-	0.588	- 0.189	0.961	6.633	10.423	2.396	4.645	4.748
*P*	-	> 0.05	> 0.05	> 0.05	< 0.05	< 0.05	< 0.05	< 0.05	< 0.05

**Table-III:** IIEF-5 scores for male kidney transplant recipients

*Group*	*Number of cases*	*Number of cases in each rating category*			
		*5-7*	*8 * *to * *11*	*12 * *to * *21*	*22* * to * *25*
ED group	63	6	25	32	-
Non-ED group	87	-	-	-	87

By comparing the ED and non-ED groups, the ED group was found to have a significantly lower score than non-ED group recipients in terms of general health, vitality, social function, emotional role function and mental health. A sizable fraction (20.6%) of the ED group showed anxiety symptoms. However, in physical functioning, physical role functioning and bodily pain, there were no differences between the two groups. ED group recipients tended to lack confidence and be fidgety, irritable, suspicious and socially withdrawn. When these recipients interact and cooperate less with their coworkers, they are relatively less successful. This also causes reduced feelings of happiness.

After communicating with ED patients, we noticed that many patients and their spouses thought that they should not be sexually active or that ED was a normal phenomenon after organ transplantation. Rebollo and colleagues showed that this was caused by psychological adaptation.^26^ Notably, we have administrated specific psychotherapy and associated medication (e.g., sildenafil) to some ED patients and asked their spouses to cooperate.^27^ After these treatments, some patients were cured or their symptoms had been alleviated. When these patients were re-evaluated with the SF-36 form and Hamilton Anxiety Scale, gradual increases of SF-36 score and Hamilton Anxiety score were found. This also helped to maintain patient compliance and stabilize graft function.

In summary, this study is one of the first studies to determine the influence of ED on the quality of life in male kidney transplant recipients. Our results highlight that ED is an important factor affecting the quality of life of male kidney transplant recipients. This suggests that more attention should be paid to overall life quality of recipients rather than just graft function. In fact, our results indicate that by treating ED and improving sexual function of male recipients, the life quality of the recipients can be enhanced. We hope this study will provide valuable information for use in follow-up treatments for male kidney transplant recipients.
